# Comparative Direct Compression Property of a Novel Pregelatinized Starch in Paracetamol Tablets

**DOI:** 10.1155/2023/5573176

**Published:** 2023-10-04

**Authors:** Tamrat Balcha Balla, Nisha Mary Joseph, Anteneh Belete

**Affiliations:** ^1^Department of Pharmaceutics and Social Pharmacy, School of Pharmacy, College of Health Sciences, Addis Ababa University, P.O. Box 9086, Addis Ababa, Ethiopia; ^2^School of Pharmacy, College of Health Sciences and Medicine, Wolaita Sodo University, P.O. Box 158, Wolaita Sodo, Ethiopia; ^3^Center for Innovative Drug Development and Therapeutic Trials for Africa (CDT-Africa), College of Health Sciences, Addis Ababa University, Addis Ababa, Ethiopia

## Abstract

**Background:**

Among all the pharmaceutical dosage forms, tablets are still the most preferred and the most commonly used option because of their advantages. The direct compression method of tablet preparation exempts several steps needed in the granulation method. Therefore, the pursuit of better direct compression tablet excipients is evident in contemporary research endeavors. Pregelatinized Taro Boloso-I starch has comparable flow properties and higher compressibility and compactibility than Starch 1500®. However, there is no evidence in the literature regarding the lubricant sensitivity and dilution potential of pregelatinized Taro Boloso-I starch. This study was aimed at performing the *in vitro* evaluation of paracetamol tablets prepared using pregelatinized Taro Boloso-I starch as a direct compression excipient using paracetamol as a model drug.

**Methods:**

Taro Boloso-I starch was pregelatinized, and its properties including amylose to amylopectin ratio, densities, flow properties, swelling power, water solubility index, particle morphology, moisture content, and moisture sorption profile were evaluated. Furthermore, the lubricant sensitivity test, dilution potential study, and compatibility test with the paracetamol drug using ATR spectroscopy were performed. The properties of the directly compressed tablets prepared accordingly were evaluated. The majority of evaluations were performed in comparison with Starch 1500®. *Results and Discussion*. PGTBIS had a significantly lower amount of amylose than Starch 1500®. In the ATR-IR spectra of the mixture of the paracetamol and pregelatinized PGTBIS, all the major absorbance peaks of the drug were maintained indicating the absence of chemical modifications. PGTBIS showed better flow properties than Starch 1500®. The modified starch was shown to withstand magnesium stearate up to 0.5% concentration.

**Conclusion:**

PGTBIS could accommodate higher drug cargo than Starch 1500® with acceptable tablet properties. Accordingly, PGTBIS starch could be taken as a potential direct compression excipient.

## 1. Introduction

While there are several types of pharmaceutical dosage forms, tablets are still the most preferred and the most commonly used option [1, 2]. The direct compression method of tablet preparation is the most desirable method. The wet granulation method has drawbacks in terms of achieving batch-to-batch reproducibility and higher productivity, especially in a low-particle size range. It is a resource-intensive process besides its stability concerns on heat and moisture-sensitive drugs.

Direct compression overcomes these problems. It is more economical as it requires fewer unit operations. It also reduces the chances of contamination and steps to be validated and documented. It avoids stability problems of especially moisture and heat-sensitive drugs, drugs whose dissolution profile is likely to change on storage, and drugs at risk of microbial growth. Furthermore, it favors faster dissolution as the tablet disintegrates directly into API particles than into granules. It reduces the wear and tear of punches due to the exemption of high compaction pressure involved in the production of tablets by slugging or roller compaction.

Flowability, low friction tendency, compressibility, and fast disintegration capacity are some of the features and criteria of directly compressible excipients [3–7]. Therefore, the pursuit of better direct compression tablet excipients is prominent in the research endeavor.

While starch is one of the leading polymers for use as a pharmaceutical excipient of several advantages [7], native Taro Boloso-I starch is reported to have appreciable compressibility and also compatibility with paracetamol [8]. However, it has poor flowability [9].

Pregelatinization enhances flow property of Taro Boloso-I starch. There is a report in the literature [10] that was compared with NTBIS and Starch 1500® in terms of the bulk density, tapped density, true density, Hausner ratio, and Carr's index, and as a result, it is considered a potential direct compression binder. In addition, compressibility/compactibility of PGTBIS is also pronounced with a Heckel yield pressure of 104.4 MPa and a tablet-breaking force of 138.0 N when 300 mg PGTBIS is compressed at 12 kN. Accordingly, it is recognized as an encouraging direct compression excipient.

There is no evidence in the literature regarding the compatibility of the PGTBIS with paracetamol, its lubricant sensitivity, and dilution potential by using specific drugs. This study was aimed at performing the in vitro evaluation of paracetamol tablets prepared using pregelatinized Taro Boloso-I starch as a direct compression excipient.

## 2. Materials and Methods

### 2.1. Materials

Taro Boloso-I was obtained from Areka Agricultural Research Institute, located at Areka (300 km south of Addis Ababa), Wolaita, Ethiopia. Pure paracetamol (China Associate Co Ltd, China) was donated by Ethiopian Pharmaceutical Manufacturing Share Company (EPHARM). Sodium hydroxide and magnesium stearate (BDH Poole Co, UK), sodium chloride (Sörensen, Leuren, Denmark), Ac-Di-Sol® (FMC, Co., USA), iodine resublimed (Reagent Chemicals Services Ltd., UK), hydrochloric acid 37% (Riedel-deHaën®, Germany), and potassium iodide (UNI-CHEM Chemical Reagents, USA) were used as obtained from the Laboratory of School of Pharmacy, Addis Ababa University.

### 2.2. Methods

#### 2.2.1. Preparation and Characterization of the Pregelatinized Starch

Taro Boloso-I starch (NTBIS) was extracted as per the methods described elsewhere in the literature [10]. Then, it was pregelatinized using a method optimized by Balla et al. [10, 11]. Accordingly, 15% (w/v) slurry of NTBIS was heated in a water bath at 66.22°C with continuous heating and uniform stirring for 20 min. The pregelatinized starches were then dried at 40°C for 48 h and powdered in a laboratory grinder (Pulverisette 2, Fritsch, Germany) and passed through a 224 *μ*m aperture sieve. Finally, the samples were stored separately in tightly sealed glass containers. The amylose to amylopectin ratio was determined by the colorimetric assay method, and the morphological study was performed using scanning electron microscopy (SEM) [12]. Swelling power, water solubility index, and moisture sorption pattern were determined by the methods by Balcha et al. and Paulos et al. [9, 13].

#### 2.2.2. Lubricant Sensitivity Study

The lubricant sensitivity study was performed using the method used elsewhere by Svačinová et al. [14]. Tablets of PGTBIS were prepared with magnesium stearate at various concentrations including 0.00, 0.25, 0.50, 0.75, 1.00, 1.50, and 2.00% (w/w). Forty grams of each of the mixtures was mixed for 5 min in a Turbula mixer (Willy A. Bachofen AG, Turbula 2 TF, Basel, Switzerland) and compressed to produce 10 mm diameter flat-surfaced 300 mg tablets at 17 kN. The compactibility was assessed by using the method used elsewhere [15, 16]. The tablet properties including the lubricant sensitivity ratios were calculated 24 hrs after compression using the following equation:

(1) $$\begin{array}{c} \text{LSR}=\left(\frac{{\text{TS}}_{0}-{\text{TS}}_{L}}{{\text{TS}}_{0}}\right), \end{array}$$

where LSR, TS0, and TBL stand for the lubricant sensitivity ratio, tensile strength of PGTBIS alone, and the tensile strength of PGTBIS mixed with lubricant, respectively.

#### 2.2.3. Dilution Potential Study

Tablets of 300 mg weight containing 20%, 30%, 40%, and 50% paracetamol were prepared using PGTBIS or Starch 1500® by the direct compression method (Table 1). In brief, paracetamol, Ac-Di-Sol®, and starch were mixed for 10 min in the Turbula mixer, and after the addition of 0.5% magnesium stearate, mixing was continued for 5 min. Paracetamol tablets were then compressed using the instrumented single-punch tablet machine (Korsch AG XP1 K0010288, Germany) at a compression force of 17 kN [17]. In all cases, the tablet properties were evaluated after 24 h of production.

#### 2.2.4. Properties of Compressed Tablets

To determine thicknesses, 10 tablets were taken and their thicknesses were measured using a sliding caliper scale (Nippon, Sokutei, Japan). The tablet bulk density was determined from the weight, thickness, and diameter data according to the methods described elsewhere [18]. To measure the tablet-breaking force (TBF), 10 tablets from each batch were taken and the average of force readings using a tablet hardness tester (CALEVA, G.B., Caleva Ltd., UK) was reported. The tensile strength was calculated from the TBF, thickness, and diameter data according to the following equation [14, 18]:

(2) $$\begin{array}{c} \text{TS}=\frac{2\text{TBF}}{\pi \text{DT}}, \end{array}$$

where TS, TBF, *D*, and *T* stand for tensile strength, the breaking strength, diameter, and thickness of tablets, respectively.

In order to evaluate the friability of compressed tablets, 20 tablets of each batch were placed into the friability tester. The friability tester was rotated for 4 min at 25 rpm, letting the tablets fall a distance of 6 inches. Then, the tablets were taken out and dusted, and the percent weight loss was calculated. The disintegration and dissolution tests were performed according to the methods described in USP-NF [19] on a disintegration tester (ERWEKA ZT504, Germany) and the type II apparatus (ERWEKA, DT600, Germany), respectively. Phosphate buffer (pH 5.8) of 900 ml medium at 37 ± 0.5°C with a stirring rate of 50 rpm was used. Five ml of aliquots was removed with blank replacement at 5, 10, 15, 20, 30, 45, and 60 min and filtered using Whatman number 1 filter paper. One ml of the filtered samples was diluted to 25 ml, and absorbance readings were taken with a spectrofluorometer CM 2203 (Solar, Belarus, Russia) at 243 nm. Phosphate buffer (pH 5.8) was used as a blank. The necessary corrections for dilution were made when calculating drug dissolution.

#### 2.2.5. ATR-IR Spectroscopy

The attenuated total reflectance (ATR)-IR spectra of pure paracetamol, PGTBIS, and paracetamol-PGTBIS physical mixture (1 : 1) were obtained with an infrared spectrophotometer (Tensor II FTIR Spectrometer, Bruker Optics, USA) in the ATR mode. For each run, 16 scans were performed in the range of a wave number of 4000–500 cm^−1^ at a resolution of 4 cm^−1^. For data presentation, Origin version 7 (Origin LabTM Corporation, USA) was applied.

#### 2.2.6. Statistical Analysis

All the results of direct measurements were presented as the arithmetic mean ± standard deviation (*π* ± *σ*). The target limit of the significance of statistical data was 95% CI.

## 3. Results

### 3.1. Amylose to Amylopectin Ratio

After the preparation of PGTBIS, its amylose and amylopectin contents were determined comparatively with that of NTBIS and Starch 1500® (Table 2).

### 3.2. Swelling Power and the Water Solubility Index

**Figure 1:**
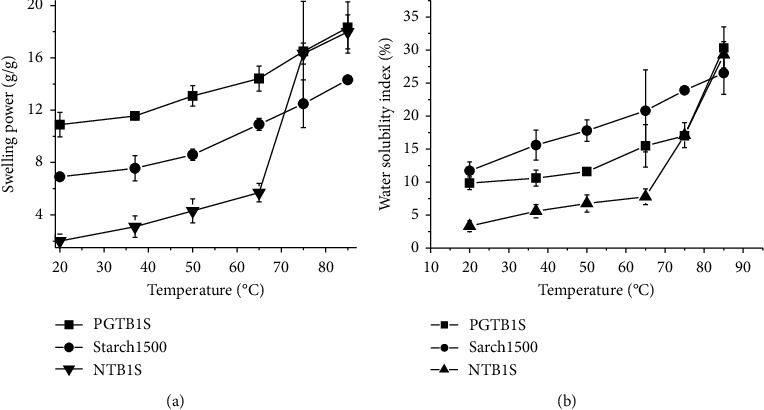
Swelling power (a) and water solubility indices (b) of PGTBIS, Starch 1500®, and NTBIS.

The swelling and solubility trends of PGTBIS, Starch 1500®, and NTBIS across 20°C–85°C are depicted graphically in Figure 1.

### 3.3. Particle Morphology

**Figure 2:**
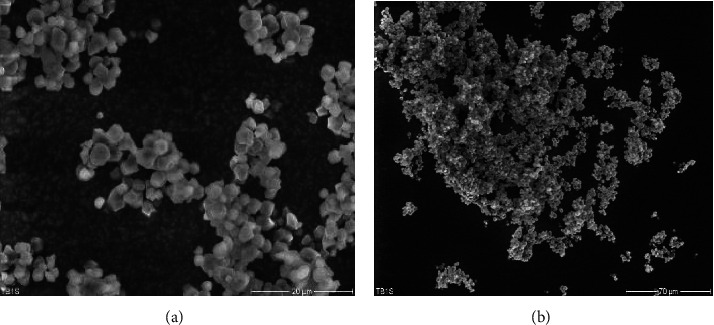
Scanning electron micrographs of PGTBIS: 20 *µ*m scale bar (a) and 70 *µ*m scale bar (b).

The scanning electron micrographs (SEM) of PGTBIS are presented in Figure 2.

### 3.4. Moisture Content

The moisture content of PGTBIS was comparatively determined with that of NTBIS. The moisture content of NTBIS, PGTBIS, and Starch 1500® were 9.11 ± 0.25%, 10.43 ± 0.42%, and 9.49 ± 0.39%, respectively.

### 3.5. Moisture Sorption Profile

**Figure 3:**
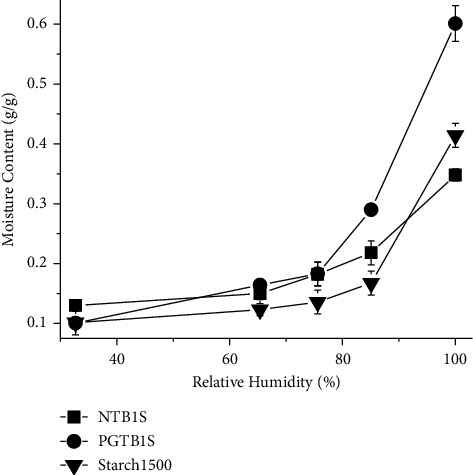
Moisture sorption isotherm of NTBIS, PGTBIS, and Starch 1500®.

Moisture sorption of starches can affect the physicochemical properties of solid dosage forms containing starches. The moisture sorption profiles of NTBIS, PGTBIS, and Starch 1500® determined at relative humidity values of 32.7%, 65.4%, 75.6%, 85.1%, and 100% by using saturated salt solutions of magnesium chloride hexahydrate, sodium nitrite, sodium chloride, potassium chloride, and distilled water, respectively, are shown in Figure 3.

### 3.6. ATR-IR Analysis

**Figure 4:**
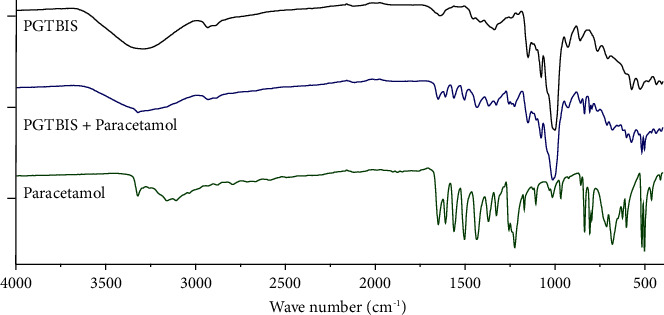
ATR spectra of pure PGTBIS, pure paracetamol, and PGTBIS to paracetamol.

To study the compatibility of the starch with paracetamol, functional groups that define paracetamol were assessed using the attenuated total reflectance (ATR) spectra of PGTBIS, 1 : 1 ratio mixture of paracetamol and PGTBIS, and pure paracetamol (Figure 4).

### 3.7. Lubricant Sensitivity

The TBF, friability, and radial tensile strength values of tablets of pure PGTBIS and lubricated at different concentrations of Mg stearate (0–2.00% w/w) were investigated (Table 3).

### 3.8. Dilution Potential

**Figure 5:**
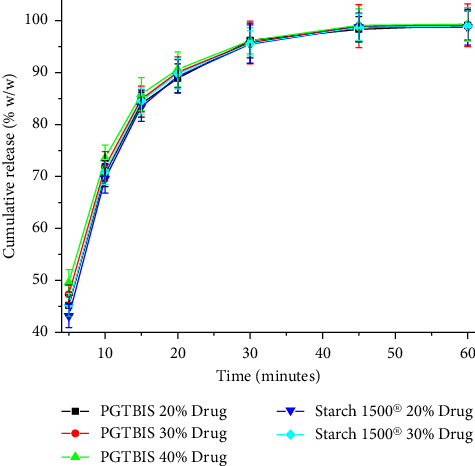
The dissolution profiles of the directly compressed paracetamol tablets prepared at different concentrations of PGTBIS and Starch 1500® with paracetamol.

The dilution potential of PGTBIS was tested in paracetamol tablets compressed at a force of 17 kN with variable concentrations of the drug at 20, 30, 40, and 50% w/w. The weight variation, TBF, TS, friability, and disintegration time were determined. Tablets of the same formulation and processes were repeated substituting PGTBIS with Starch 1500® as comparators (Table 4). Similarly, the dissolution profiles of the tablets were formulated as described in Figure 5.

## 4. Discussion

As the results show, PGTBIS had a significantly lower amount of amylose than Starch 1500®. Moreover, the process of pre/gelatinization did not have any significant effect on the amylose to amylopectin ratio of NTBIS. The reason is that pregelatinization is a physical modification and that a physical modification does not change the amylose to amylopectin ratio as described in the literature [20].

The swelling power of the three starches followed the order: PGTBIS > Starch 1500® > NTBIS at 20, 37, 50, and 65°C (Figure 1(a)). A possible explanation for the increase in the swelling power of PGTBIS than NTBIS is that the thermal disruption of crystalline phases sets starch molecules free to absorb more water molecules than amorphous intercluster lamellae [21]. However, beyond the cutoff temperature of the onset of pregelatinization, i.e., after 68.40°C [10], both PGTBIS and NTBIS had comparable swelling power. The graph (Figure 1(b)) indicates that pre-gelatinization also increased the solubility index of the starch probably due to amylose leaching [22]. The similar findings were reported elsewhere, for example, rice and corn starches [23]. At 75 and 85°C, NTBIS and PGTBIS were observed to have comparable swelling power and solubility index. In comparison with PGTBIS and NTBIS, the swelling power of Starch 1500® was, respectively, lower and higher at and below 65°C.

As it is clear from the figure, the pregelatinized starch particles had slightly smoother polygonal shapes than native granules. The morphological change might be because of partial pregelatinization that had taken place which resulted in more aggregated granules, having less physical integrity compared to NTBIS. More spherical shape, aggregation, and loss of physical integrity make changes similar to that of heat moisture-treated low amylose rice starches reported elsewhere [24, 25].

Commonly, dry starch contains 6–16% moisture. The moisture content, if high, can result in microbial deterioration of the product [26]. PGTB1S had the least moisture content (9.11 ± 0.25%), followed by Starch 1500® (9.49 ± 0.39%), and the highest (10.43 ± 0.42%) belonging to NTBIS (*p* < 0.05). This implies that the pregelatinization of NTBIS decreased the moisture content for unknown reasons, indicating its better potential stability.

The moisture sorption profile of PGTBIS was higher than the corresponding values of Starch 1500® at RH values of 65.4% and beyond (*p* < 0.05). At the RH values of 75.4% and higher, the moisture sorption of PGTBIS was higher than that of NTBIS. The likely reason for the increased moisture sorption of PGTBIS compared to that of NTBIS was the decrement of crystal phases due to hydrothermal disruption accompanied by pregelatinization [20], and it is expected for pregelatinized starches [27].

The ATR of pure PGTBIS, pure paracetamol, and PGTBIS with paracetamol in a 1 : 1 ratio (w/w) is presented in Figure 4. To ensure drug excipient compatibility, the absorbance peaks in the fingerprint region and other characteristic vibrations of paracetamol including -NH-, -OH-CO, -CH_3_, benzene ring, and phenyl-OH were considered. The peaks in the finger print region (2000–400 cm^−1^) of the mixture were coinciding with those of pure paracetamol. The sharp absorption band at 3321.29 cm^−1^ and 1650.70 cm^−1^ were corresponding to the symmetric stretching and out-of-plane (OOP) bending bands of -NH- bonds, respectively. Similarly, the strong peaks at 3164.36 cm^−1^ and 3108.69 cm^−1^ represent CO-H stretching vibration bands. The presence of a strong band in the range 3162–3035 belongs to the stretching vibration of CH_3_. The presence of aromatic rings was evidenced because there are the doublets (1562.20 cm^−1^ and 1505.14 cm^−1^), possible weak overtone, and combination bands between 2000 cm^−1^ and 1700 cm^−1^. The broad background absorption around 3350.00–3108.69 cm^−1^ (OH-stretches) with the consideration of the finger print region claiming the phenolic -OH group. The presence of the acetyl group was supported as there were strong bands at 2930.38 cm^−1^, 2887.58 cm^−1^, and 1369.60 cm^−1^ of the methyl C-H bonds. It was reinforced considering the presence of the strong protruding band at 1650.66 cm^−1^, suggesting CO stretching vibration. The presence of the peaks at 1256.90 cm^−1^ and 1224.10 cm^−1^ is common to C-O/C-N stretching vibrations [28, 29]. The presence of the vibrational absorbance bands which possibly qualify the structural groups of paracetamol implies that the chemical interaction of paracetamol with PGTBIS is unlikely [30].

The weight variation of all the tablets was quite acceptable which is below 7.5% [31]. The TBF and friability in all cases decreased and increased with the increasing concentration of the lubricant, respectively (*p* < 0.05). This is likely due to increasing bond inhibition [17]. The TBF of PGTBIS tablets without magnesium stearate, with 0.25% magnesium stearate, and with 0.5% magnesium stearate was 136.3 ± 5.1 N, 92.4 ± 4.2 N, and 75.9 ± 5.2 N, respectively. This showed the corresponding harder tablets of PGTBIS than those of Starch 1500® for which the TBF was 86.1 ± 4.0, 63.0 ± 2.6, and 56.8 ± 2.6, respectively. Tensile strengths of the tablets of PGTBIS and Starch 1500® revealed the same implications. The tensile strengths of all the tablets decreased with the addition of the magnesium stearate lubricant and increase in its concentration. The literature suggests that the optimum tensile strengths for tablets range from 0.56 to 1.12 [32]. The tensile strengths of PGTBIS exceeded this limit at and below 0.5% of the magnesium stearate concentration. This was taken as a room for the excipient to perform better when mixed with drugs of poor tabletability. In other words, it suggests that PGTBIS can be taken as a direct compression binder [33]. In all cases, whether with or without the lubricant, the tensile strengths of tablets of PGTBIS showed higher tensile strength values than those of Starch 1500®. LSR was also observed to increase with the increase in the lubricant concentration. However, for some unknown reason, the lubricant sensitivity ratio of PGTBIS was higher than that of Starch 1500®. Similarly, the percent friability values of the corresponding tablets of PGTBIS were 0.27 ± 0.01%, 0.39 ± 0.01%, and 0.56 ± 0.01%, whereas those of Starch 1500® were 0.52 ± 0.01%, 0.71 ± 0.01%, and 1.05 ± 0.01%, respectively, which again supported that the tablets of PGTBIS were more attrition resistant than those of Starch 1500® tablets prepared under the same conditions of the formulation and process factors. The increase in the concentration of MgS was shown to continuously increase the DT in all cases. This is expected due to impaired wetting by hydrophobic film formation around the particles [34]. All the tablets of both of the starches disintegrated within an acceptable time for tablets [35].

The weight variation of all the tablets was quite acceptable which was below 7.5% [31]. The TBF of the tablets prepared with PGTBIS was maintained in the acceptable range up to 30% of paracetamol, whereas the same tablets prepared with Starch 1500® were acceptable only up to 20% of the paracetamol concentration. The tensile strength of paracetamol tablets prepared with PGTBIS was observed to be >1 MPa which is an ideal target according to the established evidence [36]. According to another claim in the literature, the optimum range is within 0.56–1.12 MPa [14, 32, 37], and this is met when 40% of paracetamol is incorporated into PGTBIS. Up to 30% (w/w) dilution of PGTBIS with paracetamol, the tablets retained the quality requirements, experiencing higher dilution potential than Starch 1500®, 20% (w/w). Beyond these respective concentrations, the poor compressibility and elastic recovery of paracetamol exhibited dominance and resulted in higher friability values. The disintegration time of the tablets of paracetamol was shown to increase with the increasing concentration of the drug in the cases of both the PGTBIS and Starch 1500®.

The weight uniformity of PGTBIS-containing paracetamol tablets satisfies compendial requirements, i.e., within ±5% of the mean. The TBF of paracetamol tablets was shown to reasonably increase with the increasing proportion of the starches. The tablets of the PGTBIS paracetamol tablets were acceptably hard (TS = 1.19 MPa), up to 30% of the drug. In contrast, Starch 1500® equivalent was hard and attrition resistant enough up to only 20% (w/w). At all the paracetamol levels, the tablets of PGTBIS had higher mechanical strength (TS) than those of Starch 1500®. The paracetamol tablets of both PGTBIS and Starch 1500® beyond the respective paracetamol concentration of 30% and 20% (w/w) had low tensile strength, higher friability (>1%), or capping and lamination [38]. The DTs of paracetamol tablets in the study decreased with the increasing concentration of the drug. At all levels of the drug, PGTBIS-containing tablets disintegrated faster than that of Starch 1500®. Moreover, the tablets of PGTBIS fulfilled the requirements of fast-dissolving tablets (<3 minutes). By any means, the tablets had acceptable DT (<15 min), at all levels of the drug cargo for both types of starches.

According to the compendial requirements, the dissolution profile of paracetamol tablets is tested at a pH of 5.8. [19]. In view of that, the dissolution profiles of tablets prepared using paracetamol contents of 20, 30, and 40% (w/w) of with PGTBIS and 20 and 30% (w/w) with starch 1500® were such that all the tablets released more than 90% within 30 min. For the first 20 min, the dissolution was faster with increasing concentration of the drug for both of the starches. At and beyond 30 min, the dissolution rate was comparable for all the tablets of both of the starches, in all of the cases, perhaps because disintegration of the majority of the tablets and the subsequent dissolution that took place.

## 5. Conclusion

The findings of the present study showed that PGTBIS has comparable flow properties with standard Starch 1500®. As far as ATR spectroscopy is concerned, the pregelatinized starch did not chemically interact with paracetamol when mixed for tablet preparation. When mixed with equal respective amounts of the magnesium stearate lubricant and the paracetamol drug cargo, PGTBIS performs better than Starch 1500®. It was shown to accommodate higher drug cargo (30%) than Starch 1500® (20%) with better performance. In terms of both the lubricant sensitivity and the dilution potential, it shows higher tablet-breaking strengths and lower percent friability values. Accordingly, the PGTBIS starch could be taken as a potential direct compression excipient calling for further investigations towards its application including the brittle fracture index, Young's modulus, toughness, and stability studies both in paracetamol and other drugs.

## Figures and Tables

**Table 1:** Tablet formulation for the dilution potential study.

Ingredients	Formulations			
	1	2	3	4
Paracetamol (%)	20	30	40	50
Binding starch^*∗*^ (%)	75.5	65.5	55.5	45.5
Ac-Di-Sol® (%)	4	4	4	4
Magnesium stearate (%)	0.5	0.5	0.5	0.5

^*∗*^The formulations in the table were used in triplicates corresponding to both of the binding starches PGTBIS and Starch 1500®.

**Table 2:** Amylose and amylopectin contents of the starches.

	NTBIS	PGTBIS	Starch 1500®
Amylose	20.7 ± 1.7	20.6 ± 2.02	27.5 ± 2.6
Amylopectin	77.3 ± 4.6	77.6 ± 6.13	72.07 ± 6.5

**Table 3:** The properties of tablets of PGTBIS containing magnesium stearate (MgS) (0–2%).

^∗^MgS (%)	Starch type	Weight (mg)	^∗^TBF (N)	^∗^TS (MPa)	^∗^LSR	Friability (%)	DT (min)
0.00	PGTBIS	301 ± 7	136.3 ± 5.1	2.34 ± 0.07	0.00	0.22 ± 0.01	3.2 ± 0.2
	Starch 1500®	303 ± 14	91.1 ± 4.0	1.44 ± 0.08	0.00	0.47 ± 0.01	6.2 ± 0.2

0.25	PGTBIS	302 ± 5	92.4 ± 4.2	1.54 ± 0.08	0.34	0.39 ± 0.01	4.0 ± 0.0
	Starch 1500®	303 ± 8	63.0 ± 2.6	1.02 ± 0.03	0.29	0.71 ± 0.01	6.6 ± 0.2

0.50	PGTBIS	303 ± 6	75.9 ± 5.2	1.23 ± 0.09	0.47	0.56 ± 0.01	6.2 ± 0.3
	Starch 1500®	303 ± 8	56.8 ± 2.6	0.90 ± 0.04	0.38	1.05 ± 0.01	8.0 ± 0.2

0.75	PGTBIS	302 ± 5	54.8 ± 3.9	0.89 ± 0.07	0.62	1.09 ± 0.04	6.9 ± 0.3
	Starch 1500®	302 ± 4	55.4 ± 2.5	0.87 ± 0.04	0.40	1.20 ± 0.01	8.9 ± 0.2

1.00	PGTBIS	302 ± 4	49.2 ± 3.7	0.80 ± 0.07	0.66	1.12 ± 0.01	7.8 ± 0.4
	Starch 1500®	301 ± 6	49.8 ± 2.2	0.78 ± 0.03	0.46	1.35 ± 0.01	10.0 ± 0.2

1.50	PGTBIS	302 ± 3	33.1 ± 5.9	0.54 ± 0.09	0.77	4.70 ± 0.20	9.6 ± 0.5
	Starch 1500®	302 ± 4	32.9 ± 2.0	0.51 ± 0.03	0.65	5.30 ± 0.01	11.0 ± 0.2

2.00	PGTBIS	299 ± 3	16.6 ± 4.7	0.27 ± 0.08	0.89	8.75 ± 1.20	11.9 ± 0.5
	Starch 1500®	300 ± 4	14.0 ± 1.0	0.22 ± 0.02	0.85	Friable	13.8 ± 0.2

^*∗*^MgS, TBF, TS, and LSR stand for magnesium stearate, tablet-breaking force, tensile strength, and lubricant sensitivity ratio, respectively.

**Table 4:** Properties of tablets compressed at 17 kN of various paracetamol concentrations.

Drug (%)	Starch type	Weight (mg)	TBF (N)	TS^*∗*^ (MPa)	Friability (%)	DT^*∗*^ (min)
20	PGTBIS	302 ± 3	77.4 ± 6.1	1.34 ± 0.09	0.61 ± 0.01	2.0 ± 0.1
	Starch 1500®	300 ± 3	64.7 ± 3.1	1.10 ± 0.07	0.79 ± 0.02	5.7 ± 0.1

30	PGTBIS	300 ± 3	69.9 ± 4.2	1.19 ± 0.07	0.80 ± 0.02	1.0 ± 0.1
	Starch 1500®	302 ± 3	51.5 ± 1.8	0.87 ± 0.04	1.11 ± 0.05	4.3 ± 0.2

40	PGTBIS	303 ± 4	56.6 ± 4.2	0.95 ± 0.07	1.60 ± 0.06	0.8 ± 0.0
	Starch 1500®	301 ± 5	43.4 ± 1.7	0.73 ± 0.04	3.00 ± 0.09	3.5 ± 0.1

50	PGTBIS	301 ± 6	16.1 ± 3.2	0.26 ± 0.04	Friable	0.5 ± 0.0
	Starch 1500®	301 ± 6	15.1 ± 1.2	0.25 ± 0.03	Friable	3.0 ± 0.0

^*∗*^TS and DT stand for tensile strength and disintegration time, respectively.

## Data Availability

The data used to support the findings of this study are available upon reasonable request from the corresponding author.

## Abbreviations

BP: British Pharmacopeia
DT: Disintegration time
EPHARM: Ethiopian Pharmaceutical Share Company
LSR: Lubricant sensitivity ratio
NTBIS: Native Taro Boloso-I starch
PGTBIS: Pregelatinized Taro Boloso-I starch
TBF: Tablet-breaking force
TBIS: Taro Boloso-I starch
USP: United States Pharmacopeia.
